# Impact of the different biliopancreatic limb length on diabetes and incretin hormone secretion following distal gastrectomy in gastric cancer patients

**DOI:** 10.1038/s41598-021-02001-y

**Published:** 2021-11-17

**Authors:** Ji Yeon Park, Oh Kyoung Kwon, Jae-Han Jeon, Yeon-Kyung Choi, Ki Bum Park

**Affiliations:** 1 Department of Surgery, School of Medicine, Kyungpook National University, Kyungpook National University Chilgok Hospital, Daegu, Republic of Korea; 2 Department of Internal Medicine, School of Medicine, Kyungpook National University, Kyungpook National University Chilgok Hospital, Daegu, Republic of Korea

**Keywords:** Surgical oncology, Type 2 diabetes

## Abstract

The present study aimed to investigate changes in glucose metabolism and incretin hormone response following longer intestinal bypass reconstruction after distal gastrectomy (DG) in low BMI patients with gastric cancer and type 2 diabetes. A total of 20 patients were prospectively recruited and underwent either conventional Billroth I (BI), Billroth II with long-biliopancreatic limb (BII), or Roux-en-Y anastomosis with long-Roux limb (RY) after DG. A 75g-oral glucose tolerance test (OGTT) was given preoperatively; and at 5 days, 3 months, and 6 months postoperatively. Serum glucose, insulin, glucagon, glucagon-like peptide-1 (GLP-1), and glucose-dependent insulinotropic polypeptide (GIP) were serially measured. At 6 months after surgery, complete diabetes remission was achieved in 57.1% of the BII group but in no patients in the other two groups (p = 0.018). BII group showed a significant reduction in glucose concentration during OGTT at 6 months in contrast to the other 2 groups. In the BII group, a significant increase in GLP-1 secretion was observed after surgery but not maintained at 6 months, while postoperative hyperglucagonemia was alleviated along with a reduction in GIP. BII gastrojejunostomy with long biliopancreatic limb achieved better diabetes control with favorable incretin response after DG compared to BI or RY reconstruction.

## Introduction

Bariatric/metabolic surgery is proven effective for glycemic control in patients with type 2 diabetes (T2D) and for weight reduction. Based on mounting evidence, international guidelines for diabetes management endorse metabolic surgery as one of the main therapeutic options for diabetes management^1^. Exact underlying mechanisms for diabetes control after metabolic surgery are under investigation, including improved insulin resistance related to significant weight loss and additional mechanisms independent of weight loss, such as augmented incretin effect, alteration in bile acid metabolism, and intestinal microbiota^2,3^. However, metabolic surgery is only recommended for those with body mass index (BMI) 30 kg/m^2^ or higher. Evidence supporting surgical management of diabetes is insufficient in patients with lower BMI, and efficacy of metabolic surgery in the nonmorbidly obese population remains elusive.

Meanwhile, prevalence of gastric cancer in Korea is very high^4^. Early detection and treatment outcomes have markedly improved after implementation of a nationwide screening program^5^ and long-term quality of life has become an important issue. Although the purpose of surgical treatment is completely different for gastric cancer and bariatrics, the procedures share common features in rerouting food passage. Attempts have been made to modify conventional gastric cancer surgery to simultaneously manage gastric cancer and diabetes, recently introduced as “oncometabolic surgery”^6,7^. Further investigation into metabolic changes following oncometabolic surgery is expected to provide clues for the efficacy of metabolic surgery in the nonmorbidly obese population.

The present study aimed to investigate changes in glucose metabolism and incretin hormone response following different reconstruction methods after distal gastrectomy and the impact of different biliopancreatic limb (BPL) length on glycemic control in low BMI patients with gastric cancer and T2D.

## Methods

### Study design

This study was a prospective cohort study at a single center, Kyungpook National University Chilgok Hospital, Daegu in Korea. This study was approved by the institutional research ethics and committees of Kyungpook National University Chilgok Hospital before participant accrual (IRB File No. KNUCH 2017-07-011). All enrolled patient submitted written informed consent prior to entering the study. We performed the present study according to the Declaration of Helsinki. Participants were prospectively recruited from September 2017 to April 2019 and followed for 6 months after surgery. The study is registered on clicaltrial.gov (ClinicalTrials.gov Identifier: NCT04539769, Date of registration 07/09/2020).

### Study participants

Patients were eligible for this study if they had T2D, pathologically-proven clinical stage I gastric cancer (according to the American Joint Committee on Cancer 8th edition), and expected to undergo laparoscopic distal gastrectomy^8^. Only those with BMI < 30 kg/m^2^ were selected to evaluate surgical outcomes in the nonmorbidly obese population. Participants were considered to have diabetes if they used antidiabetic medications based on past medical history or met American Diabetes Association (ADA) definition of diabetes^9^.

Patients were excluded who had (1) baseline fasting C-peptide level < 1.0 ng/dL (possible type 1 diabetes), (2) previous radiotherapy or surgery at upper abdomen other than laparoscopic cholecystectomy, (3) other malignancies in last 5 years, (4) vulnerable conditions (pregnancy, cognitive impairment, etc.), (5) Eastern Cooperative Oncology Group (ECOG)-performance status ≥ 2, or (6) participated in other clinical trials within 6 months. Written informed consent was obtained from all participants before any study-related procedures.

### Surgical procedures

All patients successfully underwent standard laparoscopic distal gastrectomy with radical lymphadenectomy^10^. The vagus nerves were usually sacrificed during the radical lymphadenectomy.

**Figure 1:**
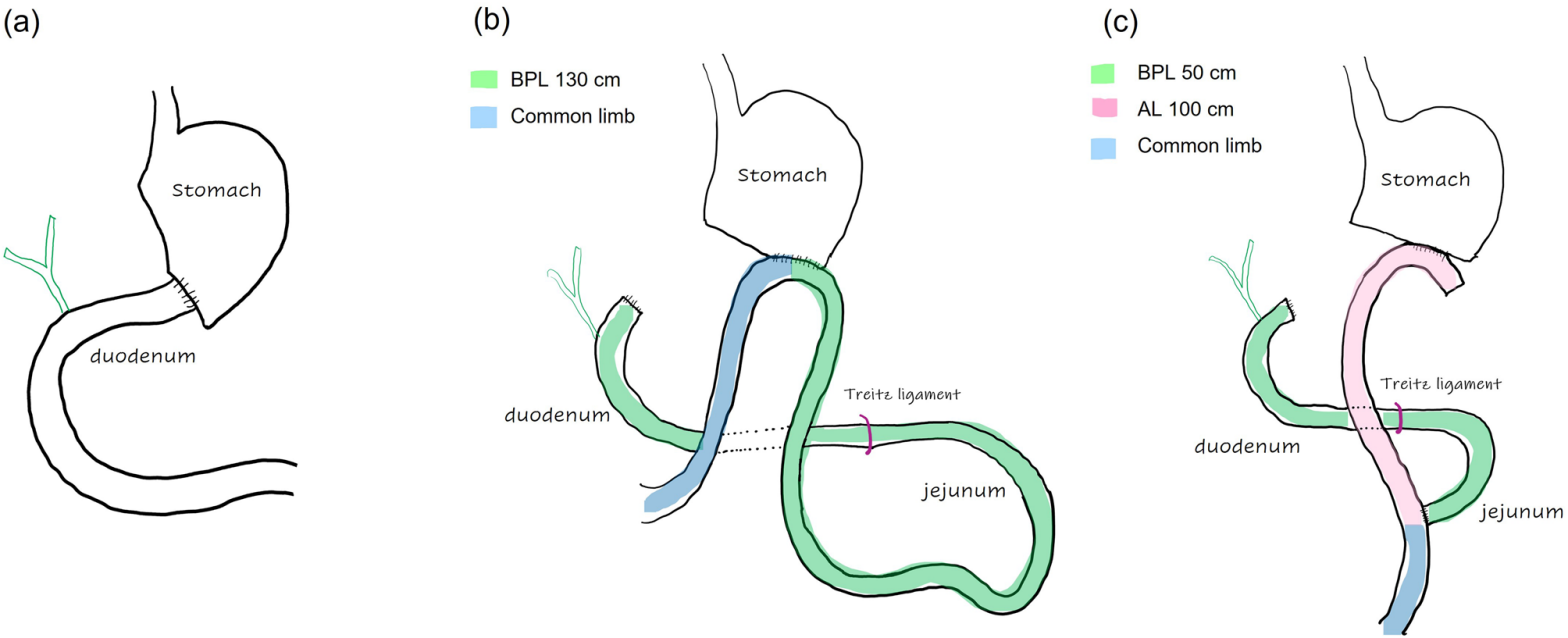
Schematics of the each reconstruction type. (**a**) Billroth I, (**b**) long-limb Billroth II, and (**c**) long-limb Roux-en-Y reconstruction (*BPL* biliopancreatic limb, *AL* alimentary limb).

Reconstruction method was selected among conventional Billroth I gastroduodenostomy (BI group), long-limb Billroth II gastrojejunostomy (BII group), and long-limb Roux-en-Y gastrojejunostomy (RY group) according to surgeons’ preference as well as the size of the remnant stomach. In long-limb BII reconstruction, the gastrojejunostomy was created using the jejunum at 100 cm distal to the Treitz ligament (the BPL length of 130 cm). In long-limb RY reconstruction, the jejunum was divided approximately 20 cm below the Treitz ligament as in the conventional manners and the distal limb was anastomosed to the remnant stomach, while jejunojejunostomy was created at 100 cm distal to the gastrojejunostomy (the BPL length of 50 cm and the alimentary limb length of 100 cm; Fig. 1).

Patients were not masked to the reconstruction type. Two designated endocrinologists followed up with all enrolled patients for 6 months after surgery and determined whether to resume antidiabetic mediations based on fasting blood glucose and HbA1c results at each visit. The endocrinologists were not blinded for the type of reconstruction.

### Follow-up

Patients were usually discharged on postoperative day 5 or 6 after diet progression. They visited the clinic 2 weeks after surgery and then at 3-month intervals within the first year. Clinical information was obtained at every visit to the outpatient clinic including body weight, BMI, and antidiabetic medication, along with biochemical results, such as fasting serum glucose, and HbA1c.

The primary endpoint was diabetes remission rate 6 months after surgery in each surgical group. Complete remission was defined as HbA1c < 6.0% and partial remission as HbA1c 6.0–6.5%; both without pharmacologic therapy according to ADA statements^11^. Patients with reduced antidiabetic medication dosages or those with decreased HbA1c levels without increasing antidiabetic medication dosage were regarded to have improvement. The secondary endpoints included dynamic changes in glucose, insulin, glucagon, and incretin hormone secretion after oral glucose challenge and changes in body weight after surgery.

### OGTT and incretin hormonal analysis

All participants were preoperatively recruited and underwent 2-h oral glucose tolerance test (OGTT) with 75 g of glucose (225 mL noncarbonated glucose drink) on the day before the surgery, on postoperative day 5 before discharge when soft diet was permitted, and at 3 and 6 months after surgery, respectively. Patients were required to fast for more than 8 h before the test, and all antidiabetic agents were omitted for 24 h before OGTT.

During OGTT, blood samples were taken before, and 30, 60, 90, and 120 min after the oral glucose loads. Retrieved samples were divided; one-half were immediately sent for conventional measurement of serum glucose level at the institutional accredited laboratory and the other half were collected in chilled EDTA tubes, which were pretreated with dipeptidyl peptidase-4 inhibitor (10 μg/ml of blood; Millipore, St. Charles, MO, USA), centrifuged at 4 °C within 30 min of collection, and stored at − 80 °C until analyses. These frozen samples were used for quantitative measurement of C-peptide, insulin, glucose-dependent insulinotropic peptide (GIP), and active glucagon-like peptide-1 (GLP-1) using the enzyme-linked immunosorbent assays from multiplexing panel according to the manufacturer’s instruction (Miliplex Human Metabolic Hormone Magnetic Bead Panel, HMHEMAG-34K).

### Statistical analysis

Statistical analysis was performed (SPSS version 25.0, SPSS Inc., Chicago, IL, USA). Categorical variables were analyzed using either Pearson’s χ^2^ test or Fisher’s exact test. Continuous variables with normal distribution were presented as mean and standard deviation, and data without normal distribution were presented as median with interquartile range. The area under curve (AUC) over 120 min during OGTT was calculated for glucose, insulin, glucagon, GLP-1, and GIP using the trapezoidal rule. Differences between the groups were analyzed using Kruskal–Wallis test, and differences between baseline and each time point after the surgery within each group were analyzed using Wilcoxon signed-rank test. P-value of < 0.05 was considered statistically significant.

## Results

### Patient characteristics and perioperative outcomes

**Figure 2:**
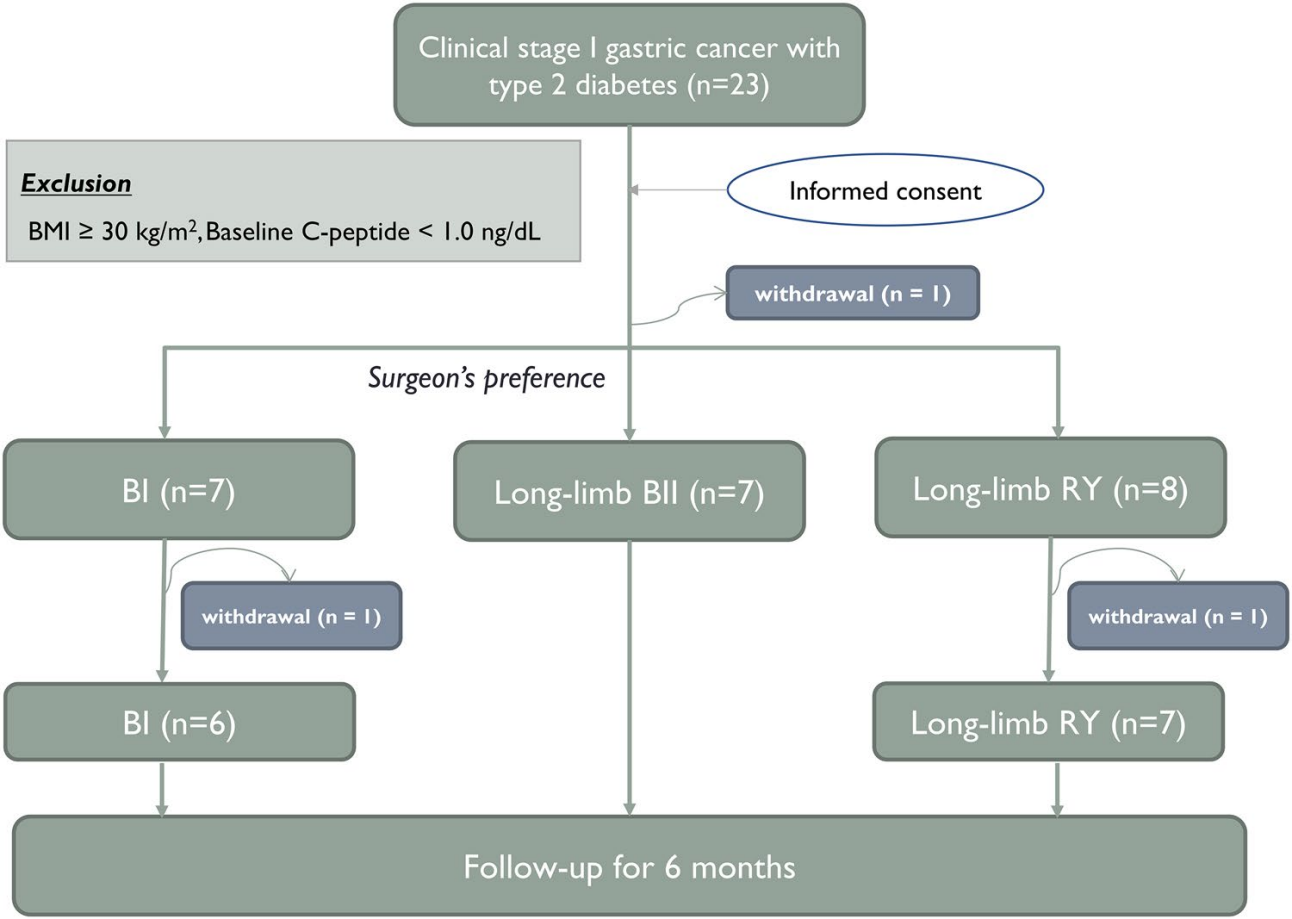
Flow diagram of the study progress (*BMI* body mass index, *BI* Billroth I, *BII* Billroth II, *RY* Roux-en-Y).

All enrolled patients were native Koreans. Of 23 patients who initially provided informed consent to participate in the study, 3 withdrew during follow-up. A total of 20 participants completed the study and were included in final analyses (6 in BI, 7 in BII, and 7 in RY group) (Fig. 2). Preoperatively, mean BMI was 25.8 ± 2.4 kg/m^2^, and mean HbA1c was 7.8 ± 1.2% (Table 1).

**Table 1:** Baseline characteristics and clinical information of the patients (n = 20).

Variables	No. of patients (%)
Age (years)	60.1 ± 10.4
**Sex**	
Male	14 (70.0%)
Female	6 (30.0%)
Body weight (kg)	67.6 ± 8.5
BMI (kg/m^2^)	25.8 ± 2.4
**Diabetes status**	
Diabetes duration (months)	36 (6–111)
**Preoperative diabetes management**	
OHA only	16 (80.0%)
Insulin use	2 (10.0%)
Newly diagnosed	2 (10.0%)
Baseline HbA1c (%)	7.8 ± 1.2
Baseline FBG (mg/dL)	121.4 ± 29.6
Baseline C-peptide (ng/ml)	3.1 (2.2–4.1)
**Surgical outcomes**	
**Reconstruction method**	
BI:BII:RY	6:7:7
Postoperative hospital stay (days)	6 (5–6)
**Pathologic stage**	
IA	15 (75.0%)
IB	4 (20.0%)
II	1 (5.0%)

Data are presented as mean ± standard deviation or medians (interquartile ranges) or number of patients (%) as appropriate.

*BMI* body mass index, *OHA* oral hypoglycemic agents, *FBG* fasting blood glucose, *BI* Billroth I, *BII* Billroth II, *RY* Roux-en-Y.

There was no postoperative complication, and all patients were discharged uneventfully. One patient in BI group was diagnosed with stage II gastric cancer in the final pathologic report and received adjuvant chemotherapy after surgery using a combination regimen of capecitabine and oxaliplatin for 6 months as recommended in gastric cancer treatment guidelines^10^. No patients experienced cancer recurrence up to 6 months of follow-up.

### Changes in body weight, BMI, nutritional status

**Figure 3:**
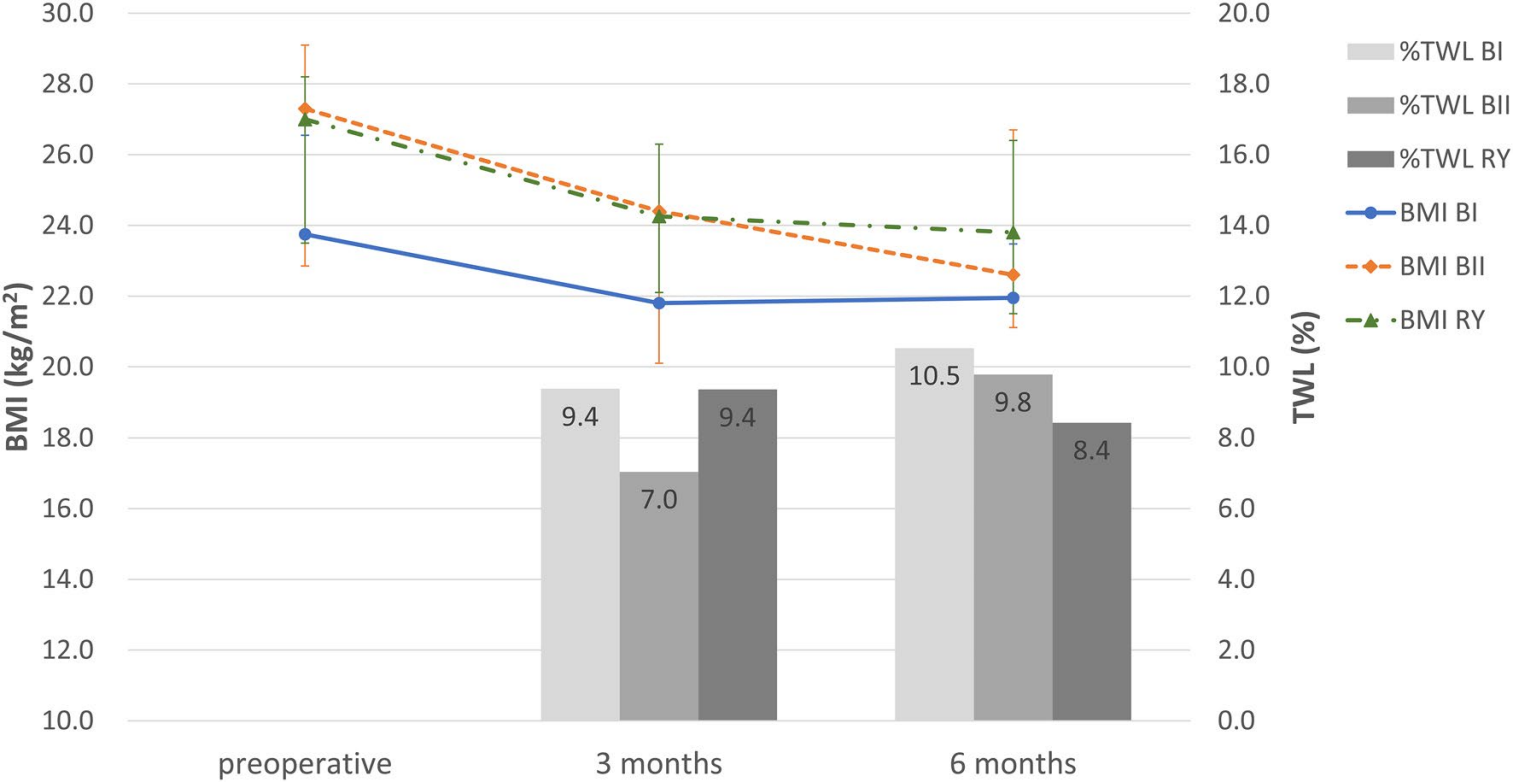
Changes in anthropometric data during the follow-up period after surgery (*BMI* body mass index, *%TWL* percentage of total weight loss, *BI* Billroth I, *BII* Billroth II, *RY* Roux-en-Y).

At baseline, there was no significant difference in BMI among the three groups (p = 0.121). Patients in all the groups showed a significant BMI decrease of median 2.3 kg/m^2^ (IQR 1.6–3.3) at 6 months after surgery compared to baseline. This corresponded to a median 9.5% (IQR 6.8–12.0) of total weight loss (%TWL), most of which occurred during the first 3 months. There was no significant difference in the amount of BMI change or %TWL between the 3 groups (Fig. 3). Although serum albumin levels significantly decreased in BI and BII group after surgery, no patient experienced hypoalbuminemia or protein malnutrition in all 3 groups (Table 2).

**Table 2:** Changes in HbA1c, fasting blood glucose and serum albumin levels after surgery.

	At baseline	At 3 months	*p*-value*	At 6 months	*p*-value^†^
**HbA1c (%)**					
BI	8.2 (7.4–9.5)	7.2 (6.6–9.3)	0.599	7.5 (6.4–8.5)	0.207
BII	6.9 (6.6–8.2)	6.2 (5.9–6.9)	0.046	6.0 (5.7–6.2)	0.046
RY	7.5 (7.3–8.6)	6.8 (6.6–7.5)	0.063	6.7 (6.1–7.9)	0.041
**Fasting blood glucose (mg/dL)**					
BI	118.0 (92.0–129.0)	145.0 (128.0–168.8)	0.075	188.5 (159.5–222.5)	0.028
BII	113.0 (88.0–138.0)	113.0 (107.0–145.0)	0.207	108.0 (99.0–129.0)	> 0.999
RY	130.0 (109.0–164.0)	151.0 (105.0–180.0)	0.204	123.0 (115.0–155.0)	0.463
**Serum albumin (g/dL)**					
BI	4.6 (4.2–4.7)	4.1 (4.0–4.6)	0.125	4.3 (4.1–4.5)	0.031
BII	4.6 (4.4–4.6)	4.3 (4.1–4.3)	0.031	4.2 (4.1–4.4)	0.031
RY	4.3 (4.2–4.5)	4.1 (4.0–4.2)	0.063	4.3 (4.1–4.5)	0.406

Data are presented as medians (interquartile rages).

*BI* Billroth I, *BII* Billroth II, *RY* Roux-en-Y.

*p-value comparing baseline with 3 months.

^†^p-value comparing baseline with 6 months.

### Effects on glycemic control

Overall, patients showed a significant decrease in HbA1c level from 7.8 ± 1.2% at baseline to 7.0 ± 1.3% during 6 months after surgery (p = 0.002). Groups did not show a significant difference in HbA1c and fasting serum glucose levels at baseline. Patients in BII and RY groups showed a significant decrease in HbA1c level during 6 months after surgery (p = 0.046 and p = 0.041, respectively), while the decrease in BI group during the same period was not statistically significant (p = 0.207; Table 2). BI group showed a gradual increase in fasting glucose level during 6 months unlike the other groups and demonstrated significantly higher fasting glucose level compared to BII or RY group at 6 months after surgery (p = 0.019).

**Figure 4:**
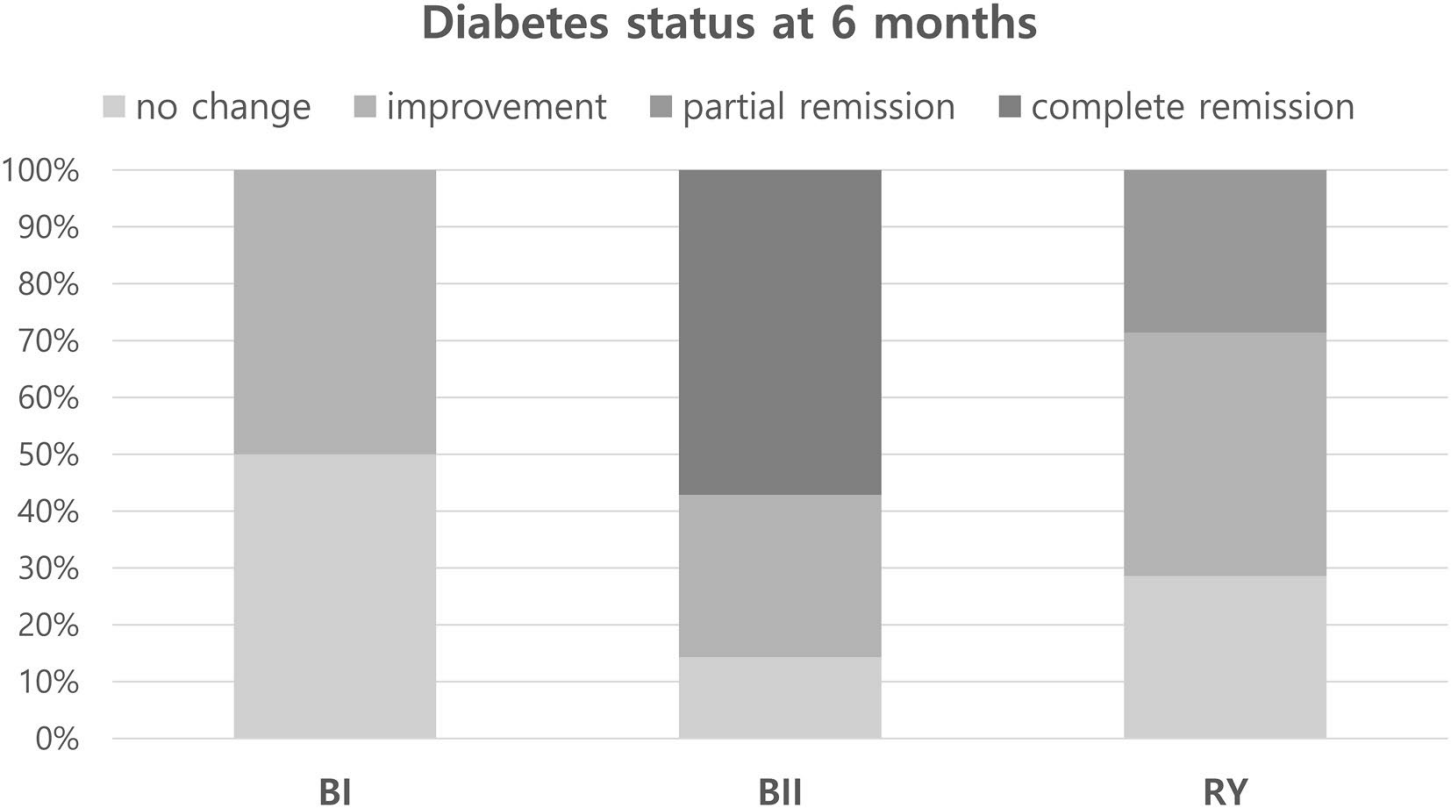
Diabetes status at 6 months after surgery.

At 6 months after surgery, 4 of 7 patients (57%) in BII group achieved complete diabetes remission and showed excellent glycemic control without antidiabetic medications but none in the other two groups (p = 0.018). None in BI group achieved remission and all were required to take oral hypoglycemic agents; 2 patients in RY group (29%) showed partial diabetes remission (Fig. 4).

### Dynamic measurements of glucose, insulin, and incretin hormones after OGTT

#### Glucose

**Figure 5:**
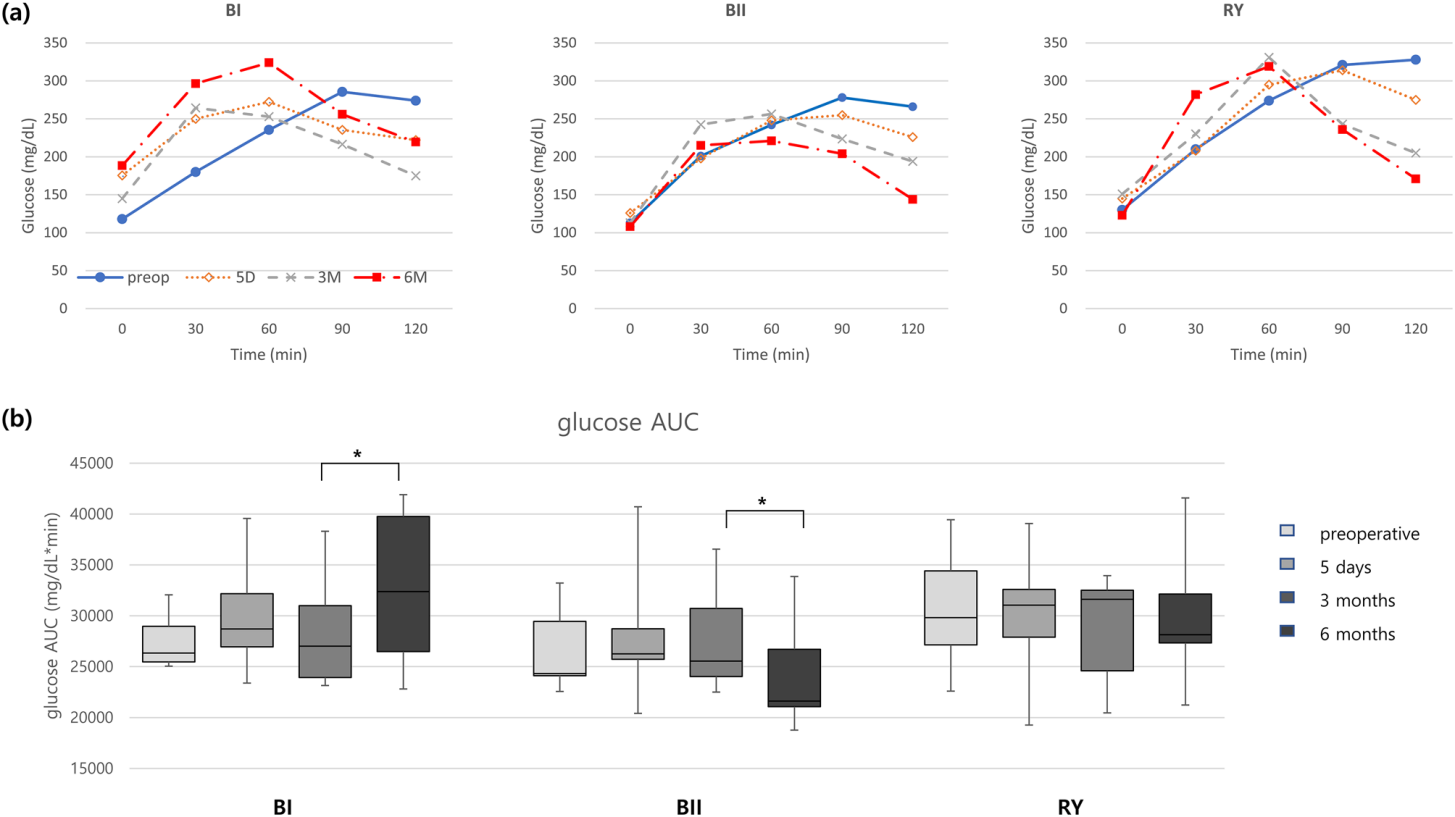
(**a**) Dynamic changes of serum glucose level after oral glucose tolerance test (OGTT) in each group. (**b**) Area under curve (AUC) during OGTT (*p < 0.05; *BI* Billroth I, *BII* Billroth II, *RY* Roux-en-Y).

Before surgery, peak serum glucose levels were not reached until more than 90 min after oral glucose challenge in all 3 groups. However, peak glucose response shifted to the left after surgery and appeared earlier (within 60 min) in all 3 groups at 6 months postoperatively. AUC of serum glucose during OGTT significantly increased in BI group at 6 months compared to that at 3 months, contrary to the significant reduction in BII group (Fig. 5 and Supplementary Table 1).

#### GLP-1

**Figure 6:**
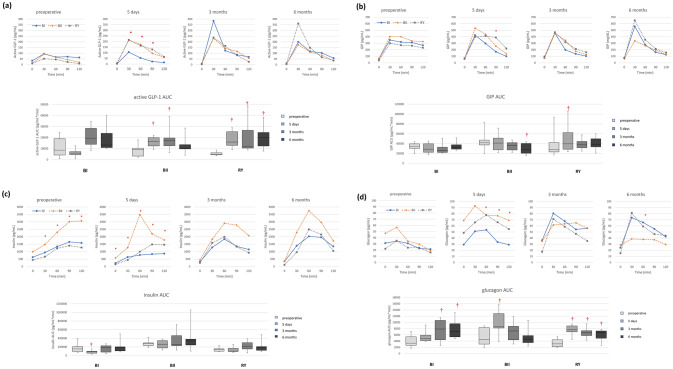
Dynamic changes and area under curve (AUC) during oral glucose tolerance test in (**a**) active glucagon-like peptide-1 (GLP-1), (**b**) glucose-dependent insulinotropic peptide (GIP), (**c**) insulin, and (**d**) glucagon (*p < 0.005 between the groups, ^†^p < 0.005 compared to the baseline value; *BI* billroth I, *BII* Billroth II, *RY* Roux-en-Y).

Active GLP-1 response to OGTT was similar between groups before surgery, showing a blunted response after glucose intake. At 5 days after surgery, a rapid increase in active GLP-1 level within 30 min after oral glucose challenge was noticed in BII and RY groups, but not in BI group. However, this intergroup difference was not maintained, and 3 groups showed similar active GLP-1 response patterns at 3 and 6 months. AUC of active GLP-1 significantly increased at 5 days and 3 months compared to baseline in both BII and RY groups. It returned to preoperative level in BII group at 6 months, contrary to RY group showing a consistently increased level (Fig. 6a and Supplementary Table 1).

#### GIP

Dynamic GIP response was similar between groups before and after surgery; all 3 groups showed a more prominent peak in response to oral glucose and returned to fasting level faster after surgery than before surgery. At 6 months after surgery, BII group appeared to show a lower GIP level compared to BI or RY group, although the difference was not statistically significant. AUC of GIP in the BII group was significantly decreased at 6 months compared to baseline, unlike BI and RY groups that showed similar AUC (Fig. 6b and Supplementary Table 1).

#### Insulin

A blunted “early” insulin response to oral glucose without a prominent peak was noted in all 3 groups before surgery (Fig. 6c and Supplementary Table 1). BII group recovered this early response immediately after surgery within 5 days, and peak appeared in 60 min. The other 2 groups showed similar changes at 3 months after surgery. However, all 3 groups showed no significant changes in insulin AUC during 6 months after surgery, except for a slight decrease on day 5 in BI group.

#### Glucagon

BII and RY groups showed a marked increase in glucagon secretion at 5 days after surgery compared to baseline, while the increase occurred at 3 months in the BI group (Fig. 6d and Supplementary Table 1). Glucagon AUC demonstrated that BI and RY groups maintained the increased status until 6 months; however, glucagon AUC in the BII group returned to baseline level in 3 months and was maintained afterward.

## Discussion

This study demonstrated that Billroth II gastrojejunostomy with 130 cm-long BPL achieved more effective diabetes control with favorable incretin response than either Roux-en-Y gastrojejunostomy with 100 cm-long alimentary limb or conventional Billroth I gastroduodenostomy after distal gastrectomy for gastric cancer. To our knowledge, this is the first study to evaluate dynamic changes in serum glucose, insulin, and glucagon levels along with incretin hormone secretion following different types of reconstruction after distal gastrectomy in patients with gastric cancer and T2D.

In this study, metabolic improvement was most prominent in the BII group with a long BPL followed by the RY group, and the least improvement in the BI group. This concurred with previous studies that advocated reconstructing with Billroth II or Roux-en-Y configuration to achieve better glycemic control after radical gastrectomy^12–14^. This suggests that nutrient bypassing duodenum and/or rapid exposure to the distal bowel can serve as the mechanism of action in improved glycemic control after gastric cancer surgery^15^. Particularly, the impact of weight loss on metabolic improvement would be less prominent in this low BMI population (BMI < 30 kg/m^2^), whose margin for weight loss was much smaller than those with morbid obesity. Therefore, mechanisms independent of weight may provide a dominant contribution to postoperative glycemic control, and one major impetus could be changes in the gut hormone secretion.

Looking deeper at dynamic changes in serum glucose, insulin, glucagon, and gut hormones during OGTT, we evaluated the impact of longer-limb bypass on the entero-insular axis after surgery. Previous studies have shown that delayed glucose peak time was correlated to decreased insulin sensitivity, increased insulin resistance, and failure of pancreatic β-cell function in patients with diabetes^16,17^. This delayed glucose peak resulted from impaired early-phase insulin secretion, which was partly attributable to the blunted incretin effect in diabetes^18^. Although all 3 groups in the present study showed an earlier glucose peak during OGTT after surgery compared to baseline, the BII group demonstrated a more gradual change toward an earlier peak (down to 30 min) together with a reduction in glucose AUC at 6 months. This suggests that BII group with long BPL had a greater potential to restore the early phase of insulin secretion after oral glucose load.

One possible cause for positive changes in glucose excursion was an augmented GLP-1 secretion in the immediate postoperative period. Patients with T2D demonstrated a blunted incretin effect due to reduced GLP-1 secretion and impaired insulinotropic effect of GIP^19,20^. BII and RY groups in the present study demonstrated increased GLP-1 response after oral glucose loads within 5 days after surgery, which occurred relatively later at 3 months in BI group. It is plausible that the GLP-1 response immediately after surgery in BII and RY groups was enhanced by excluding the duodenum and proximal jejunum, while a similar effect could be found later in BI group by rapid emptying of orally-administered glucose, as shown in sleeve gastrectomy patients without diversion^21,22^. This enhanced GLP-1 secretion is thought to have been partly reflected in the improved insulin response after oral glucose challenge, which was most prominent in BII group 5 days after surgery. At 6 months, however, the GLP-1 response attenuated in the BII group, indicating complex interactions of other physiologic factors for glycemic control in the longer term. Some previous studies have shown that the exaggerated postprandial secretion of GLP-1 gradually attenuated over time after bariatric surgery^23–25^. It might be due to weight loss and the development of compensatory mechanisms. Extensive denervation during gastric cancer surgery might have further impaired the neuroendocrine signals sustaining the augmented secretion of GLP-1. A longer-term investigation is required to elucidate whether this attenuated GLP-1 response occurs only in the BII group or also occurs in the other groups, which would further elaborate the association of BPL length and GLP-1 secretion.

The results of our current study suggest that GIP may play a role in prolonged diabetes control. GIP, which originally has glucose-dependent insulinotropic activity in healthy subjects, lacks its insulinotropic effect in patients with T2D and instead stimulates glucagon secretion^19,20^. In the present study, BII group showed a significant decrease in GIP AUC during OGTT at 6 months, which seemed to correlate with the decrease in glucagon secretion back to baseline level at 6 months after transient increase. This was unlike the other two groups that demonstrated sustained hyperglucagonemia in response to oral glucose administration. It can be postulated that a decrease in GIP response in the BII group could have stabilized postoperative hyperglucagonemia, which subsequently led to better glucose homeostasis in a longer term in BII group.

Considering that the total length of small bowel bypass before reaching common channel was similar (130–150 cm) in both BII and RY groups, the different incretin hormone secretion response in these two groups after oral glucose challenge could be attributed to the different length of BPL and presence of the Roux limb (i.e., alimentary limb). There has been a consistent effort to identify the ideal limb length to achieve the greatest metabolic effect without critical nutritional deterioration in metabolic/bariatric surgery. Some investigators argue that BPL length is important in glycemic control because BPL is completely excluded from the food transit and only carries biliopancreatic secretion^26^. However, only a few studies have focused on length of BPL, and a recent systematic review tentatively suggested positive metabolic advantages of long BPL, although that suggestion was based on insufficient research results^27^. Meanwhile, Roux limb, which serves as the primary recipient of food, may still absorb some nutrients that do not require further digestion by biliopancreatic secretion. Previous studies have shown that Roux limb became hypertrophic, with an increased number of incretin-producing cells, after Roux-en-Y gastric bypass^28^. The nutrient transit in the Roux limb might stimulate GIP secretion, consistent with the greater GIP levels observed in RY group than in BII group in this study. Based on the present study, it is suggested that the length of BPL matters more than the total length of diversion in terms of glycemic control. This is partly from favorable incretin response, but the exact underlying mechanisms require further research. Interestingly, the incretin hormonal response in each group in the present study appears to be comparable to those after popular bariatric/metabolic procedures in previous studies: BI to sleeve gastrectomy, RY to Roux-en-Y gastric bypass, and BII to biliopancreatic diversion^19,29^.

One thing to note in the present study is that postoperative changes in brain-gut signaling would be dissimilar to what we expect from metabolic surgery, as radical lymph node dissection usually accompanies extensive denervation of the upper abdomen along with vagotomy during gastric cancer surgery. Neural disruption leads to pathophysiologic changes in gastrointestinal motility as well as gut hormone secretion, and it is believed to interrupt feedback mechanisms of glucose homeostasis unlike after metabolic surgery. Historically, truncal vagotomy is known to result in hyperglucagonemia, which in turn causes glucose intolerance^30^. All the three groups in the present study initially demonstrated a paradoxical glucagon increase in hyperglycemic status after oral glucose administration. This may be partly due to vagotomy and damage to the autonomic nervous system.

The BI group in the present study demonstrated an unexpected gradual increase in the fasting glucose during the follow-up of 6 months. It is consistent with the previous study by Choi et al., which showed that BI anastomosis demonstrated less diabetes remission compared to RY reconstruction as well as a gradual increase in the fasting glucose level up to 6 months after gastric cancer surgery^31^. In the present study, the BI group demonstrated a distinct increase in the serum glucagon level, particularly at 3 and 6 months, and it can be postulated that some unknown factors produced from the food passage through the duodenum and the proximal jejunum might disturb stabilizing hyperglucagonemia. This might be one of the reasons for the gradual increase in the fasting glucose levels in the BI group. It requires further investigation to determine how vagotomy along with gastrointestinal rerouting influences the postoperative glucose dynamics after surgery in patients with gastric cancer.

Diabetes remission rate in the present study, which is 57.1% in BII group with long BPL, is still not as high as reported in bariatric/metabolic literature, and there are several possible explanations. The present study only includes patients with BMI < 30 kg/m^2^. Although many argue that metabolic surgery could be effective in these nonmorbidly obese patients, the reported diabetes remission rate tends to be lower compared to a population with higher BMI. The remission rate in the present study is comparable to the rate estimates of 42.4–43% in the previous meta-analysis, which included only studies with diabetic patients with BMI < 30 kg/m^2^^32,33^ The patients in the present study are relatively old as compared to those included in the bariatric literature, which indirectly implies worse pancreatic reserve. Asian diabetic patients are known to have markedly reduced β-cell function compared to other ethnic groups^34,35^. Additionally, the carbohydrate-rich meals favored by Koreans are thought to adversely affect glycemic control.

There are several limitations in the present study. First, the sample size is small, and the number of patients allocated to each group is even smaller. However, statistically significant differences among the three groups are noted, even with this small number of patients, and further studies are expected to reinforce the findings. Second, patients are not randomly assigned to each reconstruction group, and the baseline characteristics are not completely balanced between the groups. Third, residual gastric volume after distal gastrectomy is not considered in the final analyses. However, previous studies have shown that residual gastric volume has minimal influence on the recovery of oral diet after distal gastrectomy, and it is expected to have minimal impact on subsequent glucose metabolism after food intake^36^.

## Conclusion

BII gastrojejunostomy with elongated BPL achieves better diabetes control after distal gastrectomy compared to BI or RY reconstruction (with shorter BPL) in patients with clinical stage I gastric cancer and T2D. It is recommended that BII reconstruction with long BPL be used after distal gastrectomy in the patients with gastric cancer and T2D to simultaneously control diabetes and gastric cancer, particularly for those who are highly likely to benefit from long-limb bypass without increased risk of malnutrition. Eligible patients need to be carefully selected with consideration of a patient’s age, cancer stage, and expected survival.

## Supplementary Information

Supplementary Figure 1.

Supplementary Table 1.
