# Supplementary material for: Impact of the different biliopancreatic limb length on diabetes and incretin hormone secretion following distal gastrectomy in gastric cancer patients
Source: Sci Rep. 2021 Nov 17;11:22451. doi: 10.1038/s41598-021-02001-y (PMC8599427; doi:10.1038/s41598-021-02001-y)

Supplementary Figure 1. Dynamic changes of gut hormones during OGTT by time point in each surgery group. (a) active GLP-1, (b) GIP, (c) insulin, and (d) glucagon

(a) Active GLP-1

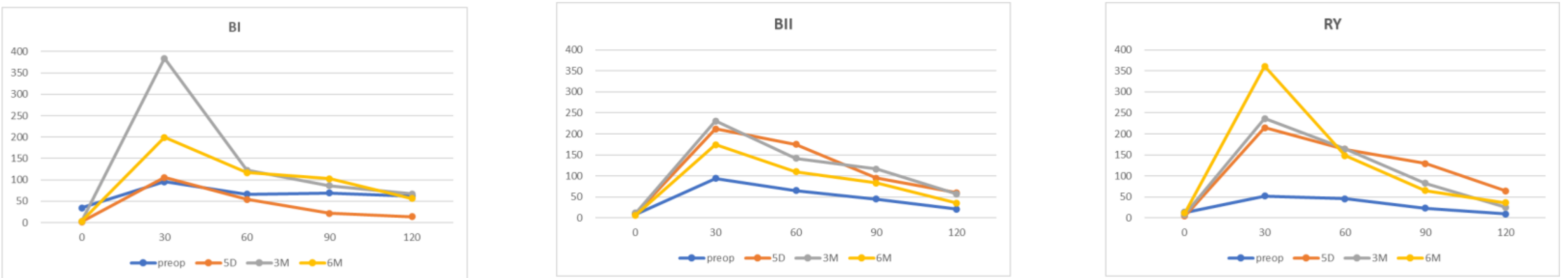

(b) GIP

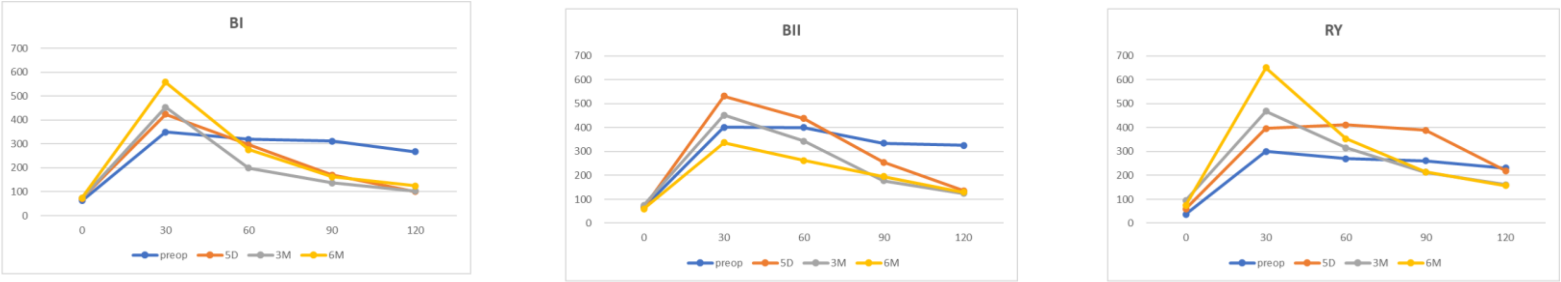

(c) insulin

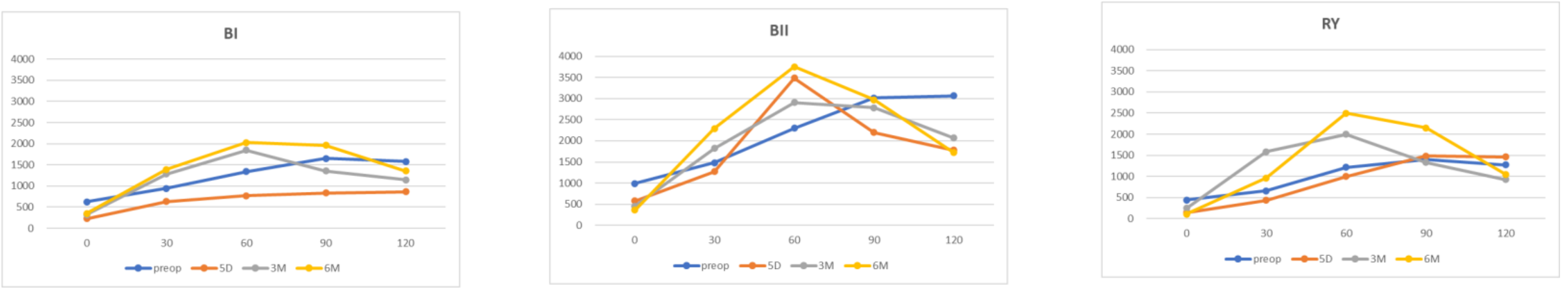

(d) glucagon

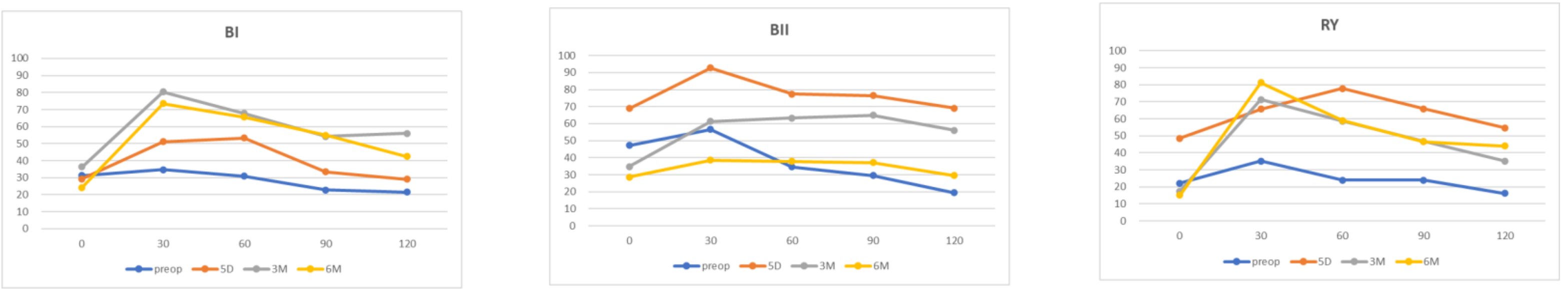

Supplement: Supplementary file 1 — Supplementary Figure 1. [file 41598_2021_2001_MOESM1_ESM.pdf]
