# Supplementary material for: Impact of the different biliopancreatic limb length on diabetes and incretin hormone secretion following distal gastrectomy in gastric cancer patients
Source: Sci Rep. 2021 Nov 17;11:22451. doi: 10.1038/s41598-021-02001-y (PMC8599427; doi:10.1038/s41598-021-02001-y)
Supplement: Supplementary file 2 — Supplementary Table 1. [file 41598_2021_2001_MOESM2_ESM.docx]

Supplementary table 1. Dynamic measurements of glucose, insulin, glucagon, and incretin hormones during the oral glucose tolerance test

|  | **Baseline** | | | | **5 days** | | | | **3 months** | | | | **6 months** | | | |
| --- | --- | --- | --- | --- | --- | --- | --- | --- | --- | --- | --- | --- | --- | --- | --- | --- |
|  | BI | BII | RY | p-value | BI | BII | RY | p-value | BI | BII | RY | p-value | BI | BII | RY | p-value |
| **Glucose** | | | | | | | | | | | | | | | | |
| Fasting (mg/dL) | 118 (92 – 129) | 113 (88 – 138) | 130 (109 – 164) | 0.562 | 176 (146 – 205) | 126 (119 – 164) | 145 (126 – 150) | 0.132 | 145 (128 – 169) | 113 (107 – 145) | 151 (105 – 180) | 0.369 | 189 (160 – 223) | 108 (99 – 129) | 123 (115 – 155) | 0.019^a^ |
| Peak (mg/dL) | 299 (255 – 320) | 287 (247 – 297) | 331 (263 – 388) | 0.158 | 278 (246 – 316) | 258 (247 – 301) | 314 (285 – 349) | 0.378 | 271 (229 – 350) | 263 (211 – 347) | 331 (255 – 339) | 0.741 | 324 (255 – 419) | 230 (206 – 309) | 319 (287 – 396) | 0.092 |
| AUC (* 10^3^ mg/dL·min) | 26.3 (25.2 – 30.4) | 24.3 (24.1 – 30.1) | 29.8 (25.8 – 34.8) | 0.294 | 28.7 (26.0 – 34.5) | 26.3 (25.5 – 20.6) | 31.0 (27.1 – 32.7) | 0.420 | 27.0 (23.2 – 33.6) | 25.5 (23.5 – 33.3) | 31.6 (20.5 – 32.7) | 0.940 | 32.4 (24.4 – 41.9) | 21.6 (20.6 – 27.2) | 28.1 (27.2 – 35.9) | 0.082 |
| **Insulin** | | | | | | | | | | | | | | | | |
| Fasting (pg/mL) | 624 (228 – 904) | 992 (487 – 1299) | 440 (240 – 569) | 0.138 | 229 (163 – 525) | 578 (421 – 899) | 147 (79 – 395) | 0.013^c^ | 318 (72 – 759) | 453 (148 – 760) | 548 (202 – 432) | 0.656 | 649 (170 – 899) | 363 (313 – 1386) | 112 (64 – 303) | 0.134 |
| Peak (pg/mL) | 1690 (1119 – 3329) | 3154 (3052 – 3733) | 1559 (1227 – 2022) | 0.014^c^ | 1017 (499 – 1358) | 3748 (2196 – 5008) | 1911 (1221 – 2879) | 0.006^a^ | 2468 (515 – 3174) | 2671 (1491 – 8037) | 3069 (1566 – 4990) | 0.509 | 2072 (1212 – 3050) | 1538 (1689 – 6376) | 2493 (1402 – 2914) | 0.313 |
| AUC  (* 10^3^ pg/mL·min) | 154.6 (81.8 – 154.6 | 276.1 (219.3 – 296.4) | 133.5 (96.4 – 167.2) | 0.018^c^ | 96.2 (41.4 – 115.8) | 264.4 (169.9 – 368.2) | 105.6 (93.9 – 200.9) | 0.007^a^ | 193.2 (47.6 – 241.8) | 263.8 (203.5 – 567.2) | 203.0 (130.1 – 307.2) | 0.199 | 192.7 (84.9 – 278.6) | 340.4 (151.3 – 400.9) | 186.7 (117.4 – 212.9) | 0.302 |
| **Glucagon** | | | | | | | | | | | | | | | | |
| Fasting (pg/mL) | 31.2 (20.2 – 64.8) | 47.2 (26.6 – 74.9) | 22.2 (16.2 – 51.8) | 0.315 | 29.3 (19.5 – 59.2) | 69.0 (46.6 – 130.4) | 48.5 (20.0 – 77.3) | 0.313 | 36.3 (13.2 – 61.6) | 34.8 (16.2 – 56.6) | 17.5 (15.1 – 43.9) | 0.773 | 24.2 (13.2 – 56.7) | 28.7 (16.9 – 39.8) | 15.1 (13.2 – 18.3) | 0.076 |
| Peak (pg/mL) | 38.7 (28.3 – 69.8) | 56.7 (33.4 – 87.8) | 35.3 (22.1 – 53.2) | 0.500 | 53.5 (50.3 – 76.7) | 92.7 (77.4 – 164.9) | 77.8 (65.9 – 77.8) | 0.133 | 88.9 (52.5 – 148.2) | 67.4 (37.8 – 89.4) | 71.3 (66.9 – 121.2) | 0.456 | 73.7 (59.1 – 120.1) | 54.0 (31.8 – 64.3) | 81.2 (51.7 – 95.6) | 0.195 |
| AUC (* 10^3^ pg/mL·min) | 3.36 (2.38 – 6.21) | 4.62 (2.74 – 8.89) | 3.10 (2.14 – 4.57) | 0.355 | 4.87 (4.06 – 6.88) | 8.71 (7.91 – 15.56) | 7.73 (6.74 – 9.20) | 0.075 | 7.88 (3.37 – 11.33) | 7.26 (3.48 – 9.78) | 6.79 (5.69 – 7.72) | 0.904 | 7.09 (4.93 – 10.95) | 4.66 (2.68 – 6.13) | 6.83 (4.63 – 7.69) | 0.208 |
| **Active GLP-1** | | | | | | | | | | | | | | | | |
| Fasting (pg/mL) | 34.6 (17.2 – 67.1) | 8.6 (2.0 – 37.6) | 13.3 (10.4 – 20.9) | 0.293 | 2.1 (1.1 – 8.2) | 10.2 (3.5 – 21.7) | 4.6 (3.0 – 17.8) | 0.133 | 3.3 (1.7 – 33.8) | 11.6 (3.1 – 22.1) | 8.4 (4.8 – 12.7) | 0.756 | 3.3 (135 – 22.8) | 6.5 (2.0 – 29.4) | 11.5 (1.5 – 22.4) | 0.843 |
| Peak (pg/mL) | 98.2 (34.6 – 305.1) | 111.7 (42.9 – 186.0) | 66.7 (59.2 – 141.2) | 0.934 | 107.8 (74.6 – 142.7) | 211.8 (149.3 – 318.5) | 215.8 (178.3 – 401.2) | 0.005^a, b^ | 383.6 (204.9 – 555.9) | 230.9 (160.3 – 413.5) | 236.6 (183.2 – 502.1) | 0.617 | 199.6 (181.3 – 434.5) | 174.1 (137.4 – 267.3) | 360.5 (153.0 – 449.6) | 0.413 |
| AUC (* 10^3^ pg/mL·min) | 8.6 (2.6 – 22.6) | 9.0 (2.9 – 10.7) | 4.9 (4.3 – 6.6) | 0.655 | 5.9 (3.5 – 8.6) | 16.3 (12.1 – 20.6) | 16.0 (12.7 – 27.9) | 0.005^a, b^ | 19.4 (12.2 – 31.1) | 17.1 (10.1 – 25.0) | 12.1 (9.0 – 27.2) | 0.906 | 13.2 (10.5 – 30.2) | 10.2 (9.5 – 16.6) | 19.8 (11.6 – 25.1) | 0.260 |
| **GIP** | | | | | | | | | | | | | | | | |
| Fasting (pg/mL) | 62.0 (53.3 – 126.0) | 62.8 (45.5 – 136.6) | 37.0 (27.0 – 117.2) | 0.552 | 71.3 (26.7 – 109.9) | 66.4 (41.6 – 79.7) | 60.0 (59.5 – 78.7) | 0.885 | 72.6 (60.9 – 79.2) | 75.1 (49.3 – 163.7) | 96.0 (83.5 – 124.5) | 0.171 | 73.7 (54.3 – 148.9) | 60.6 (47.1 – 116.2) | 75.3 (52.8 – 99.5) | 0.741 |
| Peak (pg/mL) | 357.5 (287.3 – 452.9) | 496.4 (392.4 – 515.2) | 299.7 (248.0 – 583.7) | 0.420 | 425.1 (339.8 – 567.2) | 532.3 (347.7 – 922.9) | 411.6 (334.1 – 950.6) | 0.793 | 453.7 (335.4 – 632.5) | 499.0 (401.0 – 577.3) | 468.4 (425.9 – 801.5) | 0.585 | 557.8 (428.3 – 680.8) | 355.3 (293.6 – 552.6) | 650.7 (379.9 – 899.1) | 0.194 |
| AUC (* 10^3^ pg/mL·min) | 34.7 (27.2 – 41.3) | 41.8 (36.5 – 48.1) | 28.0 (21.0 – 52.4) | 0.375 | 28.7 (20.3 – 43.4) | 41.2 (26.1 – 60.5) | 39.8 (27.0 – 80.2) | 0.473 | 26.1 (21.2 – 38.6) | 35.9 (25.4 – 43.3) | 37.8 (31.0 – 47.3) | 0.324 | 32.8 (27.4 – 42.5) | 27.9 (20.0 – 42.7) | 38.7 (30.0 – 54.9) | 0.306 |

BI, Billroth I; BII, Billroth II; RY, Roux-en-Y; AUC, area under the curve; GLP-1, glucagon-like peptide-1; GIP, glucose-dependent insulinotropic peptide

Data presented as median (interquartile ranges). Kruskal-Wallis H test with Bonferroni correction method for multiple comparisons in post-hoc analysis. P-values refer to comparing outcomes between the groups. Statistically significant in post-hoc analysis between ^a.^ BI vs BII, ^b.^ BI vs RY, ^c.^ BII vs RY. AUC calculated from time point 0 to 120 minutes for all values.
